# Core-shell cylinder (CSC) nanotemplates comprising mussel-inspired catechol-containing triblock copolymers for silver nanoparticle arrays and ion conductive channels†

**DOI:** 10.1039/c8ra00630j

**Published:** 2018-03-16

**Authors:** Hiroshi Yabu, Shusaku Nagano, Yuki Nagao

**Affiliations:** WPI-Advanced Institute for Materials Research (AIMR), Tohoku University 2-1-1, Katahira, Aoba-Ku Sendai 980-8577 Japan hiroshi.yabu.d5@tohoku.ac.jp; Venture Business Laboratory, Nagoya University Furo-Cho, Chikusa-Ku Nagoya 464-8603 Japan; School of Materials Science, Japan Advanced Institute for Science and Technology (JAIST) 1–1 Asahidai Nomi Ishikawa 923-1292 Japan

## Abstract

Catechol moieties, which are found in mussel-adhesive proteins, allow the interaction of various kinds of materials that results in substantial adhesion to a wide variety of materials and in the reduction of metal ions to solid metals. Various types of catechol-containing polymers mimicking adhesion and reduction properties have been reported, however, due to its reactivity to a wide variety of functional groups, only a few reports about the formation of block and sequence controlled copolymers containing catechol groups. This is the first report about the synthesis of triblock copolymers containing catechol groups by reversible-addition fragmentation transfer (RAFT) polymerization. The synthesized triblock copolymer forms a core–shell cylinder (CSC) phase-separated structure, in which PVCa domains located the surface of cylinders, and it works as a template for silver nanoparticle arrays and a proton conductive channel. Since triblock copolymer has broader latitude to form phase-separated structures, the triblock copolymer containing catechol groups can be suitable for templates of inorganic nanoparticle arrays.

## Abstract 2

This is the first report on the synthesis of triblock copolymers containing catechol groups and it forms a core–shell cylinder phase, which can work as a template for Ag nanoparticle arrays and proton conduction.

## Graphical abstract

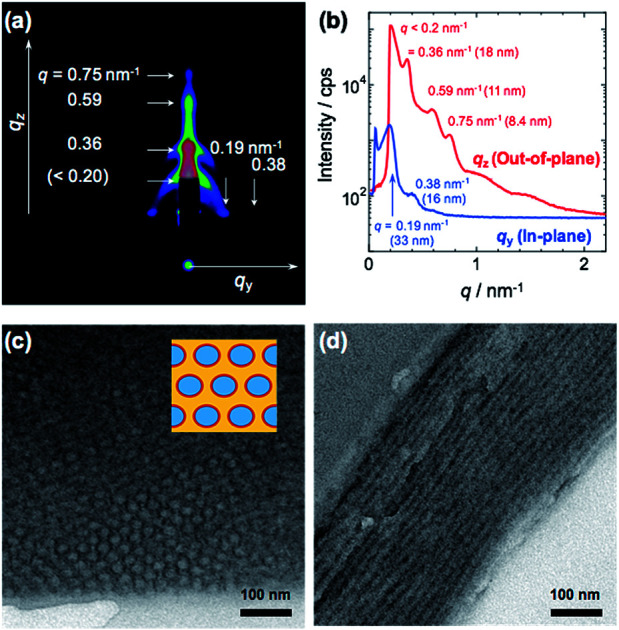

## Introduction

Catechol moieties, which are found in mussel-adhesive proteins, facilitate the interaction of various kinds of materials *via* hydrogen bonding, chelation, π–π stacking and the Michael addition reaction, resulting in strong adhesion to a wide variety of materials.^1^ Based on this variety of interactions between catechol groups and material surfaces, the underwater adhesion properties of catechol-containing polymers have been well investigated over the past decade.^2–8^ Furthermore, since the phenolic hydroxyl group has a reductive property that can reduce metal ions to solid metals, catechol groups can be used as reductants to form metal nanoparticles.^9–12^ Various kinds of catechol-containing polymers possessing adhesion and reduction properties have been reported.^4,13–18^ However, due to their reactivity to a wide variety of functional groups, there have only been a few reports on the formation of block and sequence controlled copolymers containing catechol groups,^19^ which requires propagation reactions without side reactions in order to achieve living polymerization conditions.

We first reported the synthesis of poly(vinyl catechol-*block*-styrene) (PVCa-*b*-PSt) diblock copolymers by using the reversible-addition fragmentation transfer (RAFT) polymerization of 3,4-dimethoxystyrene (DMSt) and styrene (St) followed by deprotection of the methoxy groups by BBr_3_ treatment.^20^ This diblock copolymer forms inverse micelles in hydrophobic organic solvents. Silver nanoparticles can be formed inside the inverse micelles by adding silver ions without any reductive agents. The reductive property has also been demonstrated in a thin film of PVCa-*b*-PSt where silver nanoparticle arrays aligned with the PVCa phases inside the film were formed.^21^ These results indicate that PVCa-*b*-PSt can be applied as a template for the formation of metallic nanostructures.

Some other examples for synthesizing diblock copolymers containing PVCa derivatives have been reported to control the architecture of monomer sequences and the resulting phase-separated structures. For example, Müller *et al.* reported a gradient monomer sequence containing catechol moieties by anionic polymerization reactions.^22^ Kim *et al.* also synthesized diblock copolymers containing PVCa moieties for lithographic templates.^23^ However, the synthesis of multiblock copolymers containing PVCa moieties and controlling their phase-separated structures are still challenging.

Since triblock copolymers form more complicated phase-separated structures than those of diblock copolymers depending on the composition of the polymer segments, it is of interest to synthesize triblock copolymers containing catechol groups. In this report, we describe the synthesis of poly(methyl methacrylate-*block*-vinyl catechol-*block*-styrene) (PMMA-*b*-PVCa-*b*-PSt) by RAFT polymerization of MMA, DMSt and St followed by deprotection of the methoxy groups. Phase-separated structures and the automatic introduction of silver nanoparticles inside the films were studied. Furthermore, proton conductivity in their thin films was investigated.

## Experimental

### Synthesis of PMMA-*b*-PVCa-*b*-PSt

#### Materials

Methyl methacrylate (MMA, ≥99.0%), styrene (St, ≥99.0%), anhydrous 1,4-dioxane (≥99.0%), boron tribromide (BBr_3_, ≥99.5%), 2,2-azobis(isobutyronitrile) (AIBN, ≥98.0%), tetrahydrofuran (THF/GR), dichloromethane (GR), and methanol (GR) were purchased from Wako Pure Chemical Industries, Ltd. Technical grade 3,4-dimethoxystyrene (99%) and 2-cyano-2-propyldodecyltrithiocarbonate (CPDTTC, 97%) were purchased from Sigma-Aldrich, USA. The inhibitor in St was removed by alumina column chromatography followed by drying with molecular sieves 3/4 Å overnight. AIBN was recrystallized from methanol and dried *in vacuo* before use. All other reagents were used as received.

#### Synthesis of triblock copolymer

**Scheme 1:**
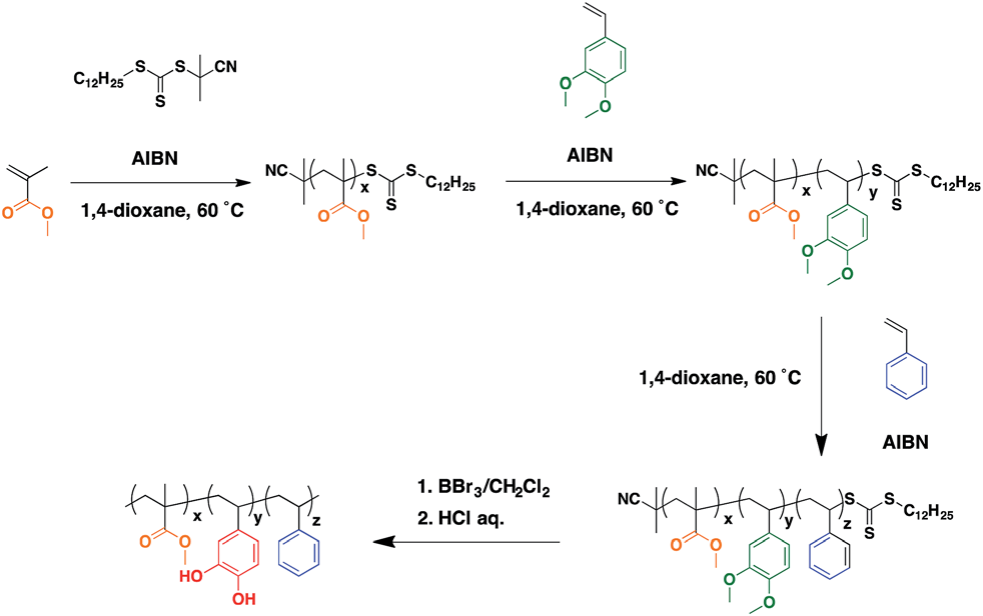
Synthetic rote of PMMA-*b*-PVCa-*b*-PSt.

Scheme 1 shows the reaction scheme for the triblock copolymer synthesis. MMA (2.00 g, 20 mmol), CPDTTC (41.6 mg, 120 × 10^−3^ mol) and AIBN (10.8 mg, 66 × 10^−3^ mmol) were dissolved in a glass tube with 1,4-dioxane (1 g). The glass tube was sealed after four freeze–pump–thaw cycles, and then placed in an aluminum block heater at 60 °C for 6 h to conduct the reversible-addition fragmentation transfer (RAFT) polymerization. The polymerization process was stopped by immediate cooling of the glass tube in liquid nitrogen and the resulting solution was reprecipitated in a large amount of methanol. The yellow precipitate was collected by centrifugation and dried *in vacuo*. PMMA-RAFT (632.5 mg), DMSt (2.00 g, 15 mmol) and AIBN (6.90 mg, 42 × 10^−3^ mmol) were dissolved in a glass tube with 1,4-dioxane (1 g) and RAFT polymerization was conducted for 6 h as in the synthesis of PMMA-RAFT. The yellow product was reprecipitated in methanol and dried *in vacuo*. PMMA-*b*-PDMSt-RAFT (856.4 mg), St (2.06 g, 20 mmol) and AIBN (5.2 mg, 32 × 10^−3^ mmol) were dissolved in a glass tube with 1,4-dioxane (1 g) and RAFT polymerization and purification were conducted as in the synthesis of PMMA-RAFT. The molecular weight of the synthesized polymer was determined by gel permeation chromatography (GPC, HLC-8320GPC, Tosoh) and ^1^H-NMR analysis (400 MHz, Bruker). The obtained molecular weight values are shown in Table 1. Actual data of GPC chromatograms and ^1^H-NMR spectra are provided in the ESI, S1–S4.†

**Table 1:** Composition of synthesized polymers

Polymer	*M*_n_ (GPC) kg mol^−1^	*M*_w_ (GPC) kg mol^−1^	*M*_w_/*M*_n_ (GPC)	*M*_w_ (NMR) kg mol^−1^	PMMA^b^	PDMSt^b^ (PVCa)	PSt^b^
**PMMA-RAFT**	11.5	12.9	1.12	17.6	171	0	0
**PMMA-*b*-PDMSt-RAFT**	17.2	19.4	1.13	33.2	171	98	0
**PMMA-*b*-PDMSt-*b*-PSt-RAFT**	27.0	30.6	1.14	60.1	171	98	259
**PMMA-*b*-PVCa-*b*-PSt**	—a	—a	—a	57.4	171	98	259

a GPC of PMMA-*b*-PVCa-*b*-PSt was not measured due to adhesive property of PVCa segment and aggregation property.

b The last three columns are repeating unit numbers of PMMA, PDMSt and PSt units calculated from ^1^H-NMR results, respectively.

#### Deprotection of PDMSt segment

PMMA-*b*-PDMSt-*b*-PSt was treated with BBr_3_ to convert the methoxy groups to hydroxyl groups. The triblock copolymer (150 mg) and dichloromethane (5 mL) were added to a glass vial, and then the vial was sealed with a rubber septum. After the solution was cooled to 0 °C, dichloromethane solution containing BBr_3_ (0.5 mL, 1.00 M) was slowly added with vigorous stirring. The solution was stirred at room temperature for 12 h. The mixed solution was added dropwise to a large volume of aqueous 1 M HCl. After stirring for 3 h, the white precipitate was collected by centrifugation and dried under vacuum. The sequence was repeated twice. The ^1^H-NMR spectrum of the deprotected polymer are provided in ESI, S5.† A signal attributed to methoxy protons of catechol moieties at *δ* = 3.75 ppm was disappeared that supported the formation of hydroxyl groups. Also, formation of hydroxyl groups was confirmed by Fourier-transform nearinfrared (FT-IR, FT/IR-6700, Jasco) spectroscopy (ESI, S6†).

#### Film preparation and silver nanoparticle formation

PMMA-*b*-PVCa-*b*-PSt was dissolved in THF to prepare a 10 wt% solution. The THF solution was cast onto a 20 mm × 10 mm quartz substrate and dried at room temperature. The prepared film was immersed in 200 mM AgNO_3_ aq. for 2–14 h to form silver nanoparticles. After immersion, the film was washed with Milli-Q membrane filtered water 3 times and dried at room temperature. UV-Vis absorption spectra of films before/after immersion in AgNO_3_ aq. were measured with a UV-vis spectrophotometer (V-670, Jasco, Japan).

#### TEM observation

To observe the interior microphase-separated structures of the films, the PVCa segment was stained with 0.2 wt% OsO_4_ aq. for 2 h. The stained film was placed on an epoxy resin substrate, and then embedded and cured in epoxy resin (EPOC, Ohken Shoji) at 70 °C overnight. The cured sample was sliced into an ultrathin specimen whose thickness was 100 nm by using an ultramicrotome (U-8, Lica). The ultrathin specimen was placed on a Cu grid with a colloidal membrane and observed by transmission electron microscopy (TEM, H-7650, Hitachi).

#### Grazing-incidence small-angle X-ray scattering (GI-SAXS)

GI-SAXS measurements for structural analysis of the microphase-separated structures were performed with a NANO-Viewer X-ray diffractometer (Rigaku) using Cu Kα radiation (*λ* = 0.154 nm) as an X-ray source and an imaging plate (Fujifilm) for detection. The GI sample stage was set using a goniometer and vertical stage. 2D GI images were recorded with X-ray incidence angles adjusted between 0.16° and 0.18°, which is between the critical angles of the films. An image was obtained by exposure for 8–24 h. The diameter of the pinhole slit-collimated X-ray beam was in the range of 0.3–0.6 mm. The camera length was set to 955 mm.

#### Proton conductivity measurement

Impedance measurements of the thin films were conducted at 40–95% relative humidity (RH) using an impedance/gain-phase analyzer (SI1260, Solartron Analytical) and a dielectric interface system (1296, Solartron Analytical). The RH and temperature were controlled using a humidity- and temperature-controlled chamber (SH-221, Espec Corp.). Porous gold paint (SILBEST No. 8560, Tokuriki Chemical Research) was used to form electrodes for impedance measurement of the films. The electrode configuration was selected to obtain measurements of current flow in the plane parallel to the substrate surface.

## Results and discussion

### Morphologies of microphase separated PMMA-*b*-PVCa-*b*-PSt thin films

As shown in Table 1, RAFT polymerizations of PMMA, PMMA-*b*-PDMSt diblock copolymer, and PMMA-*b*-PDMSt-*b*-PSt triblock copolymer were successfully conducted and yielded homo and block copolymers with narrow polydispersity indices (PDIs). After deprotection, the PDMSt was also successfully converted to PVCa, which was confirmed by ^1^H-NMR analysis (see ESI, S4†). It is noteworthy that the methyl ester groups of PMMA moieties remain after the deprotection treatment with BBr_3_. Thus, the hydrophilic PVCa moieties formed at the central block of the ABC type triblock copolymer.

**Fig. 1:**
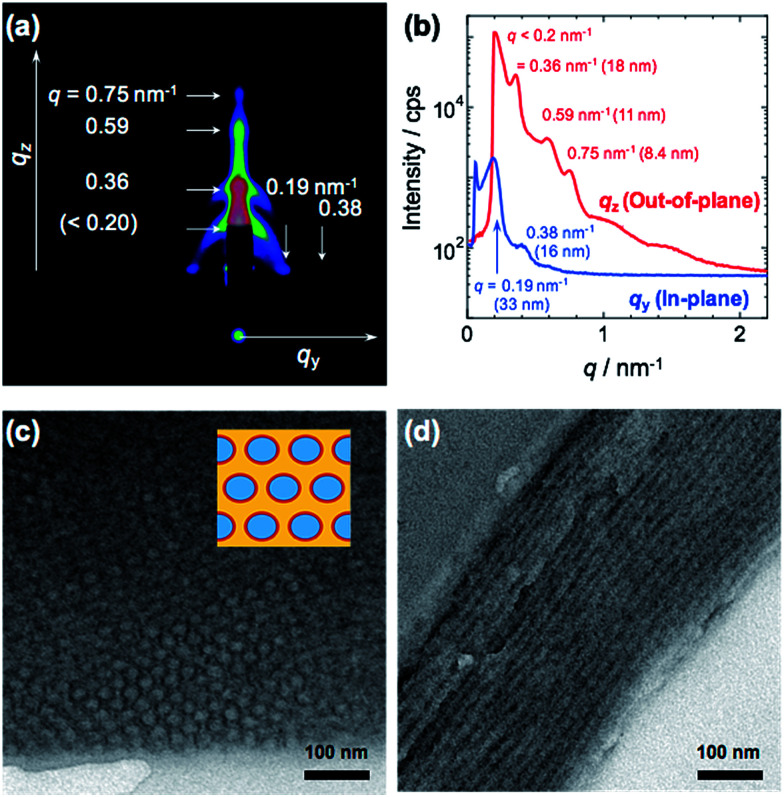
2D GI-SAXS image (a) and in-plane/out-of-plane profiles of GI-SAXS (b), cross-sectional TEM images of PMMA-*b*-PVCa-*b*-PSt triblock copolymer thin film (c), (d), respectively. The inset image of (c) shows schematic illustration of CSC phase (blue: PSt, red : PVCa, yellow: PMMA).

2D, in-plane and out-of-plane GI-SAXS results obtained from the PMMA-*b*-PVCa-*b*-PSt triblock copolymer film are shown in Fig. 1(a) and (b). Both in-plane and out-of-plane scattering peaks were clearly observed. In the in-plane scattering, the 1st scattering peak at *q* was observed at 33 nm, and the 2nd scattering peak was half the *q* value of the 1st scattering. In the out-of-plane scattering, multiple peaks were observed. The 2nd scattering peak at *q* = 0.36 nm^−1^ was attributed to 18 nm. The ratio of *q* value of these scattering peaks is nearly 1 : √3. These results indicate that a distorted cylinder phase was formed inside of the film.^24,25^

Fig. 1(c) and (d) show cross-sectional TEM images of stained PMMA-*b*-PVCa-*b*-PSt triblock copolymer thin film. Darker regions are attributed to PVCa domains stained with OsO_4_ and PMMA, and PSt domains have brighter contrast. From these images, both hexagonally arranged circles and stripe patterns were imaged. Since the darker regions are attributed to PVCa domains, the phase formed inside of the film was cylinder phase aligned along to the surface of the substrate, and PVCa domains covered the surface of respective cylinders. From the copolymerization ratio, the core part of the cylinder was comprised of PSt segments, and the PVCa segments surrounded the cores. PMMA segments filled the space among the cylindrical tubes, which means that core–shell cylinder (CSC) phase was formed. These TEM observation results are identical with the model derived from GI-SAXS measurement. Moreover, from the ^1^H-NMR measurements, the volume fractions of PMMA, PDMSt and PSt were 0.26, 0.27 and 0.47, respectively. The resulted cylinder phase, in which PSt cylinder covered with PVCa phase embedded in PMMA matrix, is identical with the core–shell cylinder (CSC) phase of ternary phase diagram that has been previously reported in the simulation of triblock copolymers by Shi *et al.*^26,27^ According to the annealing experiment in 120 °C for 12 h *in vacuo*, the CSC structure formed in the triblock copolymer was stable. This stability may be induced by intermolecular hydrogen bonding among catechol groups reported previously.^30^

### Silver nanoparticle formation

**Fig. 2:**
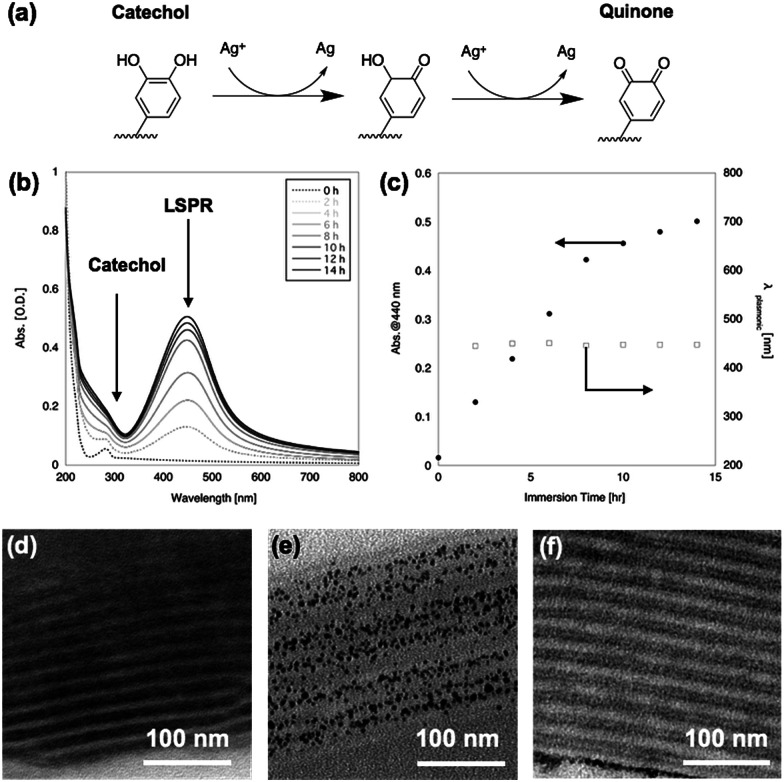
Scheme of silver ion reduction (a), UV-Vis spectra of PMMA-*b*-PVCa-*b*-PSt triblock copolymer thin film before and after immersion in AgNO_3_ aq. (b), plot of absorption peak at 440 nm (c), cross-sectional TEM images of PMMA-*b*-PVCa-*b*-PSt triblock copolymer thin film before (d) and 12 h after (e) immersion in AgNO_3_ aq. and iron chloride solution (f), respectively.

After immersion in AgNO_3_ aq., the film changed from transparent to yellow. Fig. 2(b) shows UV-vis spectra of the PMMA-*b*-PVCa-*b*-PSt triblock copolymer thin film before and after immersion in AgNO_3_ aq. The original film has an absorption peak at 280 nm attributed to the aromatic absorption of catechol moieties. After immersion in AgNO3 aq., a broad peak around 440 nm appeared and increased in intensity with increasing immersion time. The emerging peak strongly suggested the formation of silver nanoparticles inside the film since the specific absorption band was identical to the localized plasmonic absorption (LSPR) of silver nanoparticles.^28^ On the other hand, the peak at 280 nm became broad and was hidden behind the Rayleigh scattering from the film. The catechol moieties were converted to quinone groups due to the reduction of silver ions to solid silver nanoparticles (Fig. 2(a)), which also supports the formation of silver nanoparticles.

Fig. 2(c) shows a plot of the absorption value at 440 nm (solid circles) and wavelength of LSPR peak (open squares) against immersion time in AgNO_3_ aq. The plot shows that the LSPR absorption increases with increasing immersion time but the gradient changed at 8 h immersion, which indicates that the formation of silver nanoparticles decreased at this time. This may indicate that all the catechol groups were transformed into quinone groups while converting the silver ions to solid silver nanoparticles. It is noteworthy that the peak position did not change with increasing immersion time, which indicates that the size of the nanoparticles remained constant. These results strongly imply the formation of uniformly-sized silver nanoparticles and that the phase-separated structures worked as a template for the formation of silver nanoparticles while limiting their growth.

Fig. 2(d) and (e) shows cross-sectional TEM images of PMMA-*b*-PVCa-*b*-PSt triblock copolymer thin film before and after immersion in AgNO_3_ aq., respectively. Before immersion, a cross-section sliced along the long axis of the cylinders was imaged, in which stained PVCa domains were seen as striped patterns. After immersion in AgNO_3_ aq. for 2 h, uniformly-sized silver nanoparticles aligned with the PVCa phases were formed inside the film. Since the PVCa domains covered the PSt cylinders, there were two characteristic spacings among the stripes, originating from inter- and intra-cylinder areas. As shown in Fig. 2(f), a double-spaced structure was clearly imaged after immersing the film in an aqueous solution of iron chloride. It is well-known that iron ions cross-link catechol moieties due to coordination with catechol hydroxyl groups, thus the contrast in the cross-sectional TEM image was induced by iron atoms.^29^ These results indicate that silver ions transported along PVCa channels in the CSC phase and formed nanoparticles due to the reducing properties of the catechol moieties. The monomodal absorption peak of plasmonic absorption also supports the formation of uniformly-sized silver nanoparticles. These results indicate that silver ions diffused inside the PVCa domains of the PMMA-*b*-PVCa-*b*-PSt triblock copolymer thin film and catechol moieties reduced silver ions to solid silver nanoparticles when the films were immersed in silver nitrate solution. Thus, the PMMA-*b*-PVCa-*b*-PSt triblock copolymer thin film works as a template for three-dimensionally arranged silver nanoparticle arrays.

### Proton conductivity

Since the silver ions can diffuse into the PVCa domains,^30^ an ionic conduction is expected in the PMMA-*b*-PVCa-*b*-PSt triblock copolymer thin film. Proton conductivity is important for both basic understanding of ionic conductivity in polymeric materials and practical applications in the field of polymer electrolyte fuel cells. We previously reported that PVCa-*b*-PSt thin films showed proton conductivity in the range of 10^−5^ S cm^−1^ at highly humid conditions (relative humidity > 90%).^30^ In the study, we found that the size of the nanoscale channel structure did not change even though water was taken up in the films and this caused the confined water inside the channels to form hydrogen bonds with the catechol moieties, enhancing proton conductivity. Since this confinement effect should strongly depend on the size of the proton conductive channels, it is worthwhile to measure the proton conductivity of the PMMA-*b*-PVCa-*b*-PSt triblock copolymer thin film.

**Fig. 3:**
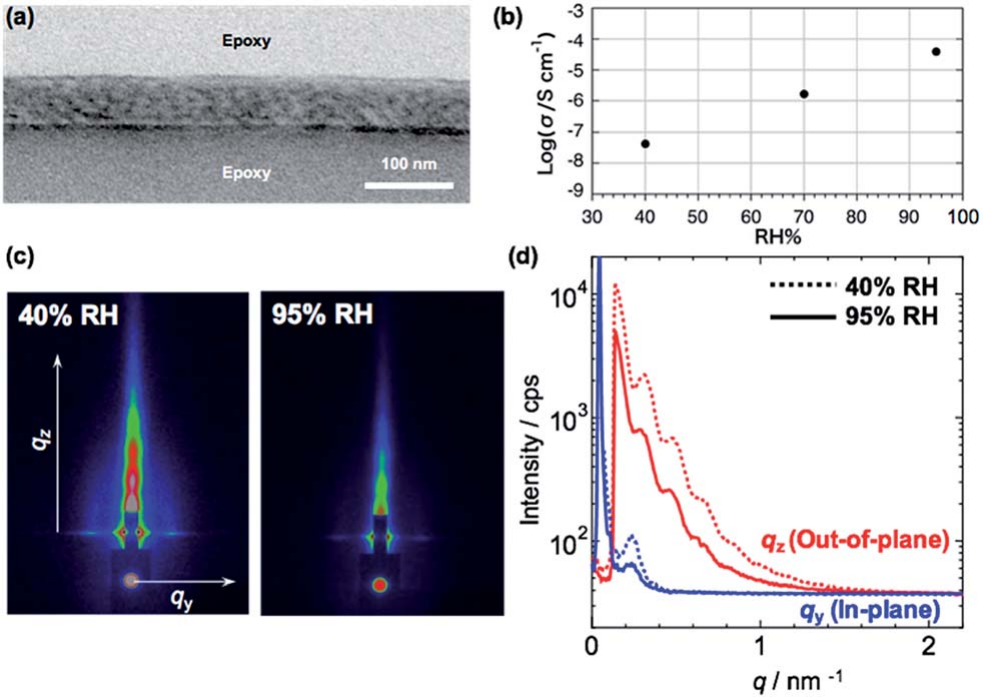
Cross-sectional TEM image of triblock copolymer (a), proton conductivity in terms of RH (b), 2D GI-SAXS images (c) and in-plane/out-of-plane profiles of triblock copolymer thin films at low (40%, dashed line) and high (95%, solid line) RH (d), respectively.

Fig. 3(a) shows a cross-sectional TEM image of PMMA-*b*-PVCa-*b*-PSt thin film used for proton conductivity measurement. Distorted CSC phase was observed with skin layers at the top and bottom surfaces. From the 2D GI-SAXS image (Fig. 3(c) left) and in-plane/out-of-plane GI-SAXS profiles of the film (Fig. 3(d)) at low relative humidity (RH 40%), the periodicity of the film was slightly shortened due to the thin film confinement but the structure is identical with the thicker film. Fig. 3(b) shows proton conductivity change with changing relative humidity. The proton conductivity of the film was less than 10^−7^ S cm^−1^ at 40% RH, the conductivity increased with increasing the relative humidity, and finally, it reached 10^−4^ S cm^−1^ at RH 95% in spite of weak acidic property of phenolic hydroxyl groups in the catechol moieties. From GI-SAXS measurement of the PMMA-*b*-PVCa-*b*-PSt triblock copolymer thin film before and after annealing in the highly humid conditions (90% RH), the channel size of the film did not change by changing the humidity (Fig. 3(c) and (d)). This result is identical with previously measured diblock case and it indicates that tiny PVCa channels filled with uptaken water molecules, which induced highly hydrogen bonded structures inside of the channels and high proton conductivity was achieved.^30^ In this experiment, the proton conductivity of the film without immersion in AgNO_3_ aq. has been measured in order to evaluate the effect of phase separated structure of triblock copolymer on the proton conductivity comparing with previously reported diblock copolymer cases. Thus, in the proton conductive experiment, hydroxyl groups in the triblock copolymer thin film have still exited. From the proton conductivity measurement of triblock copolymer thin film, the narrower PVCa conductive channels eventually induces higher proton conductivities in the thin film than that of diblock copolymer case.

## Conclusions

While there are some reports on the synthesis of triblock copolymers containing catechol groups by using a polymeric reaction, this is the first report on the synthesis of triblock copolymers containing catechol groups by living radical polymerization.^31^ The synthesized triblock copolymer forms a cylindrical phase-separated structure, in which PVCa domains are located on the surface of the cylinders. The triblock copolymer works as a template for silver nanoparticle arrays and a proton conductive channel. Since the triblock copolymer has broad latitude to form phase-separated structures, the triblock copolymer containing catechol groups is suitable for templates of inorganic nanoparticle arrays. Furthermore, the triblock copolymer films offer a new concept for proton conductive materials, which may be applicable to polymer electrolyte fuel cells.

## Conflicts of interest

There are no conflicts to declare.

## Supplementary Material

RA-008-C8RA00630J-s001
