# Supplementary material for: Core-shell cylinder (CSC) nanotemplates comprising mussel-inspired catechol-containing triblock copolymers for silver nanoparticle arrays and ion conductive channels
Source: RSC Adv. 2018 Mar 16;8(19):10627–32. doi: 10.1039/c8ra00630j (PMC9078898; doi:10.1039/c8ra00630j)
Supplement: RA-008-C8RA00630J-s001 [file RA-008-C8RA00630J-s001.pdf]

Electronic Supplementary Material (ESI) for RSC Advances.
This journal is © The Royal Society of Chemistry 2018

Electronic Supplementary Information (ESI)

Core-Shell Cylinder (CSC) Nanotemplates Comprised of Mussel-Inspired Catechol-Containing Triblock Copolymers for Silver Nanoparticle Arrays and Ion Conductive Channels

Hiroshi Yabu[†, *], Shusaku Nagano[‡], Yuki Nagao[§]

[†]WPI-Advanced Institute for Materials Research (AIMR), Tohoku University, 2-1-1, Katahira, Aoba-Ku, Sendai 980-8577, Japan

[‡]Venture Business Laboratory, Nagoya University, Furo-Cho, Chikusa-Ku, Nagoya 464-8603, Japan

[§]Graduate School of Materials Science, Japan Advanced Institute for Science and Technology (JAIST), 1-1 Asahidai, Nomi, Ishikawa 923-1292, Japa

Appendix

S1. GPC chart of synthesized polymers ……………………………………………………2
S2. ^{1}H-NMR chart of PMMA-RAFT…………………………………………………….3
S3. ^{1}H-NMR chart of PMMA-*b*-PDMSt-RAFT…………………………………………4
S4. ^{1}H-NMR chart of PMMA-*b*-PDMSt-*b*-PSt-RAFT……………………………..5
S5. ^{1}H-NMR chart of PMMA-*b*-PVCa-*b*-PSt-RAFT……………………………………6
S6. FT-IR spectrum of PMMA-*b*-PVCa-*b*-PSt-RAFT……………………………………7

Figure S1 shows GPC chromatogram of synthesized polymers. The chromatogram shows molecular weights of synthesized polymers were monodisperse and gradually increased with elongating the polymer segments.

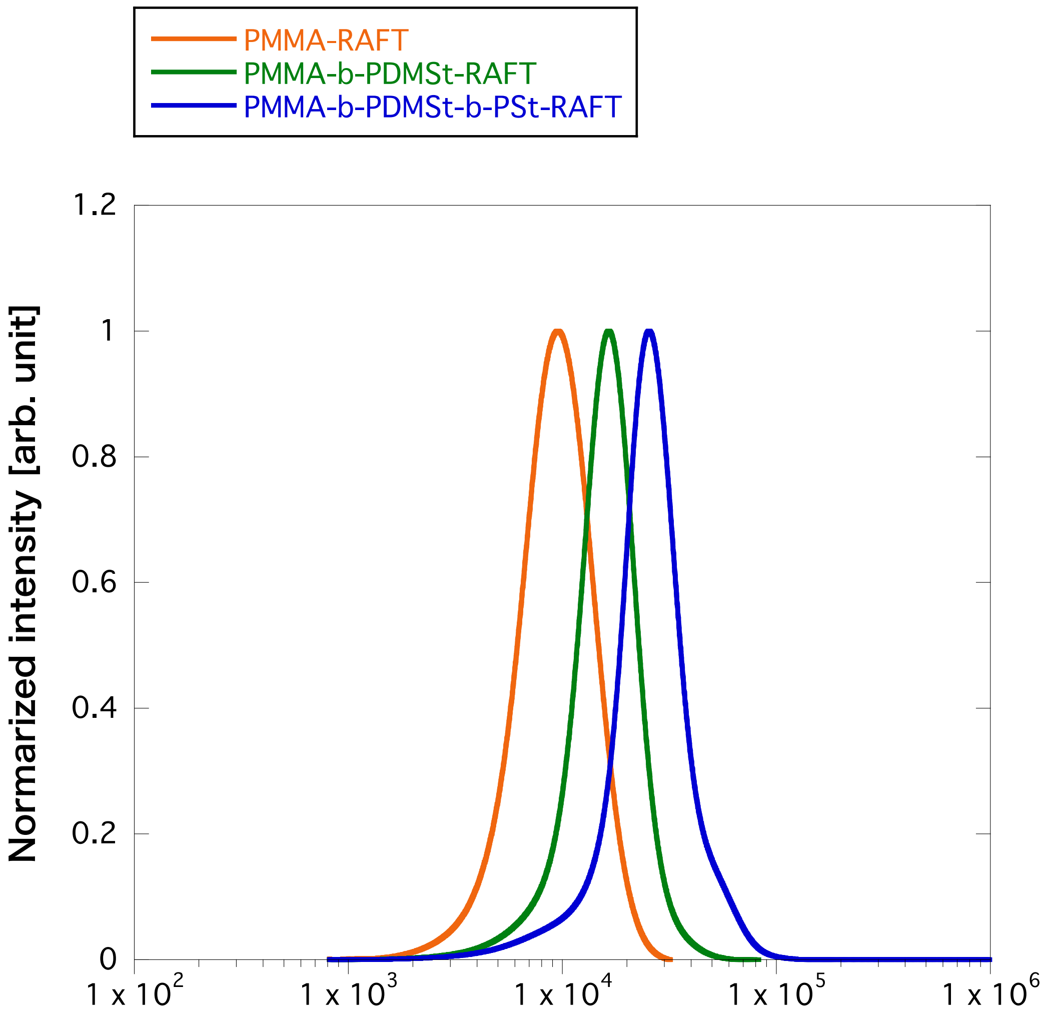

Figure S1. GPC chromatogram of PMMA-RAFT, PMMA-*b*-PDMSt-RAFT, and PMMA-*b*-PDMSt-*b*-PSt-RAFT. Horizontal axis shows molecular weights.

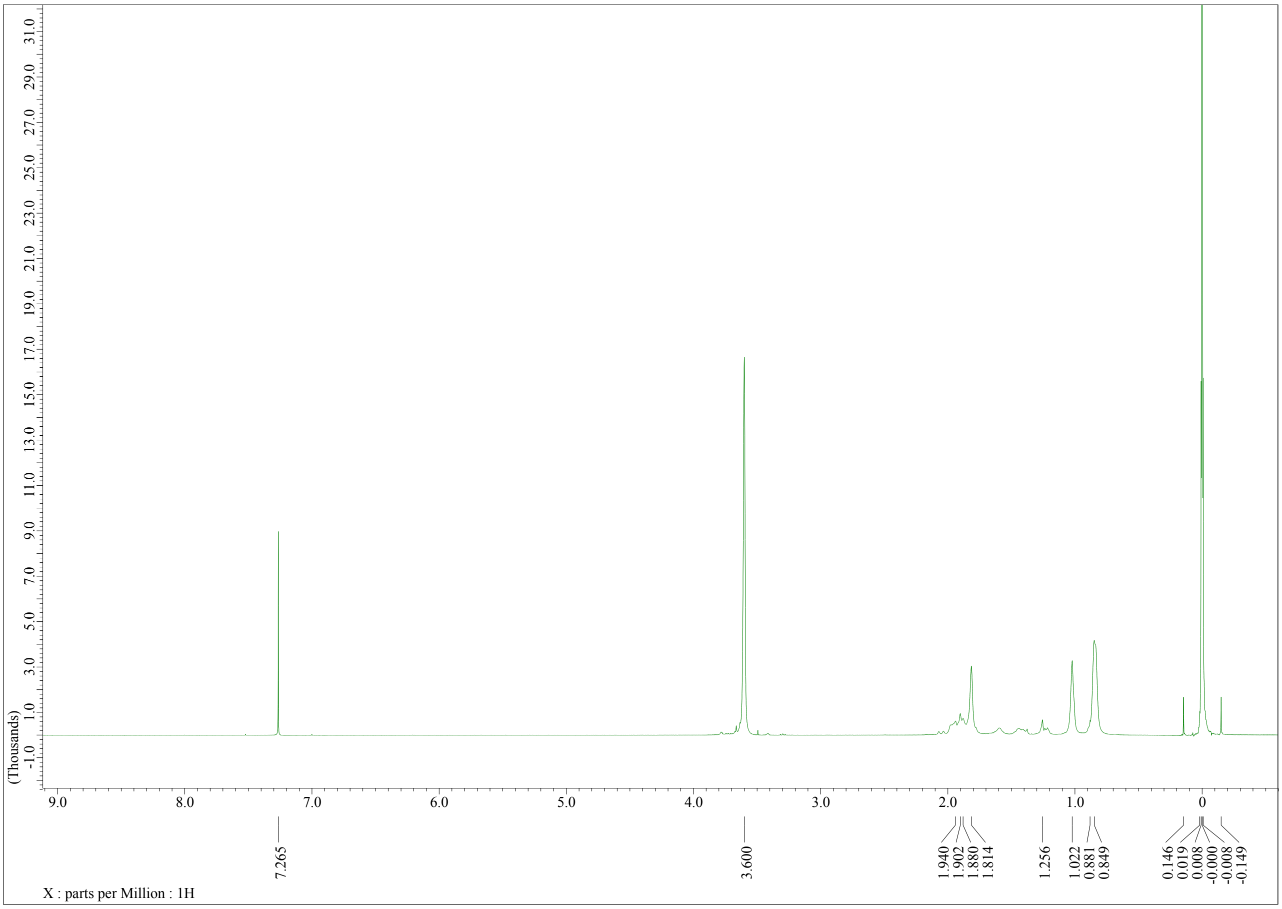

Figure S2. ^{1}H-NMR chart of PMMA-RAFT ($CDCl_3$).

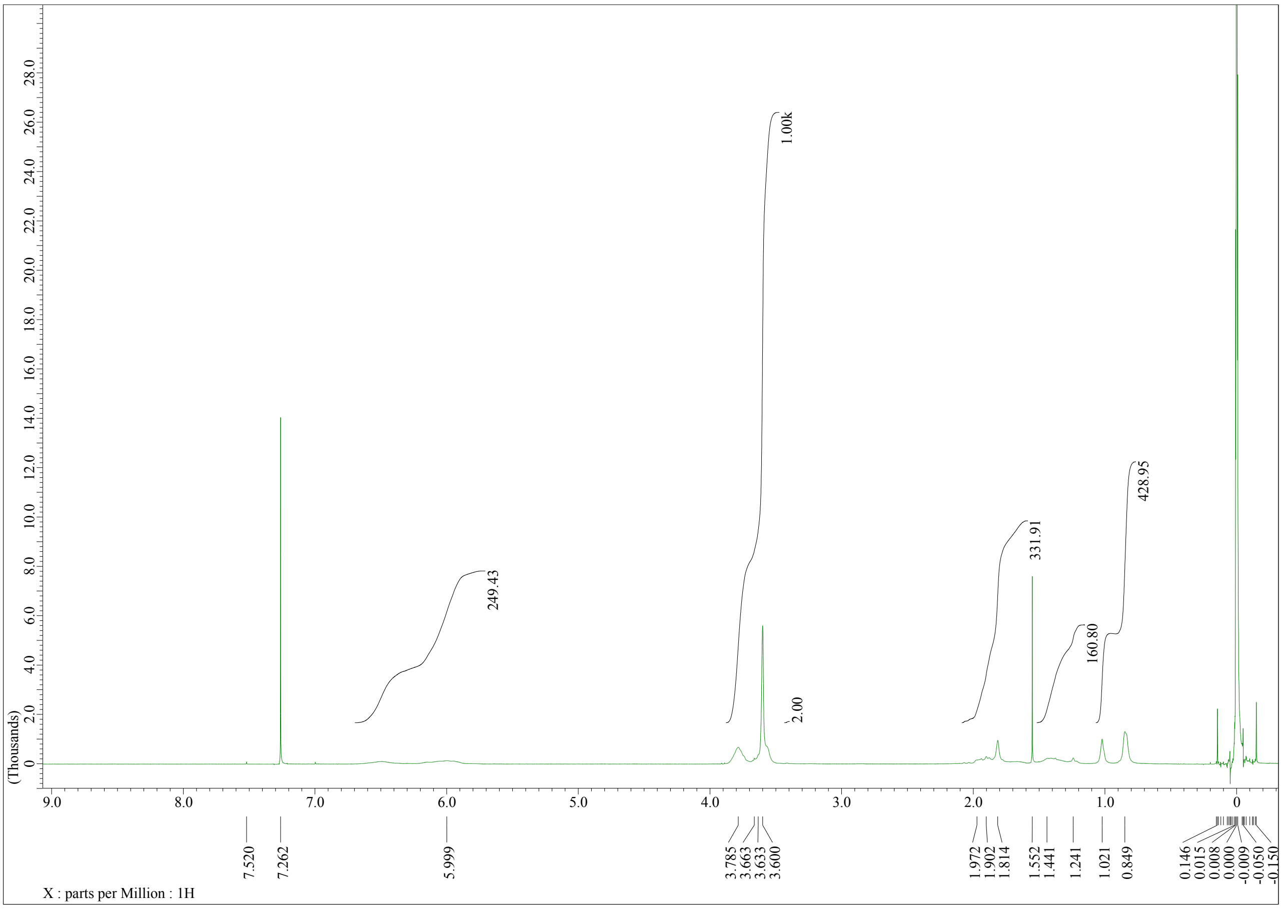

Figure S3. ^{1}H-NMR chart of PMMA-*b*-PDMSt-RAFT ($CDCl_3$)

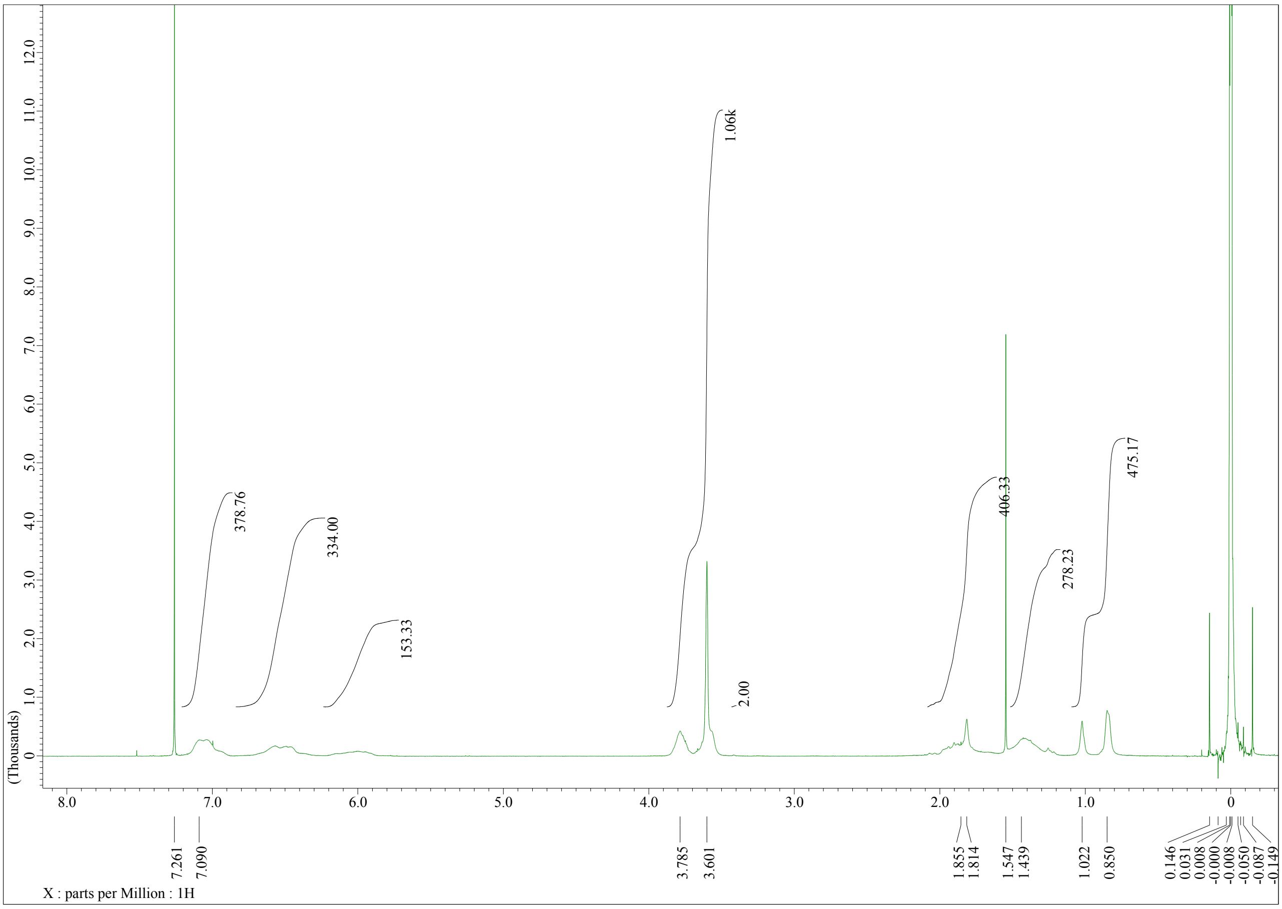

Figure S4. ^{1}H-NMR chart of PMMA-*b*-PDMSt-*b*-PSt-RAFT ($CDCl_3$)

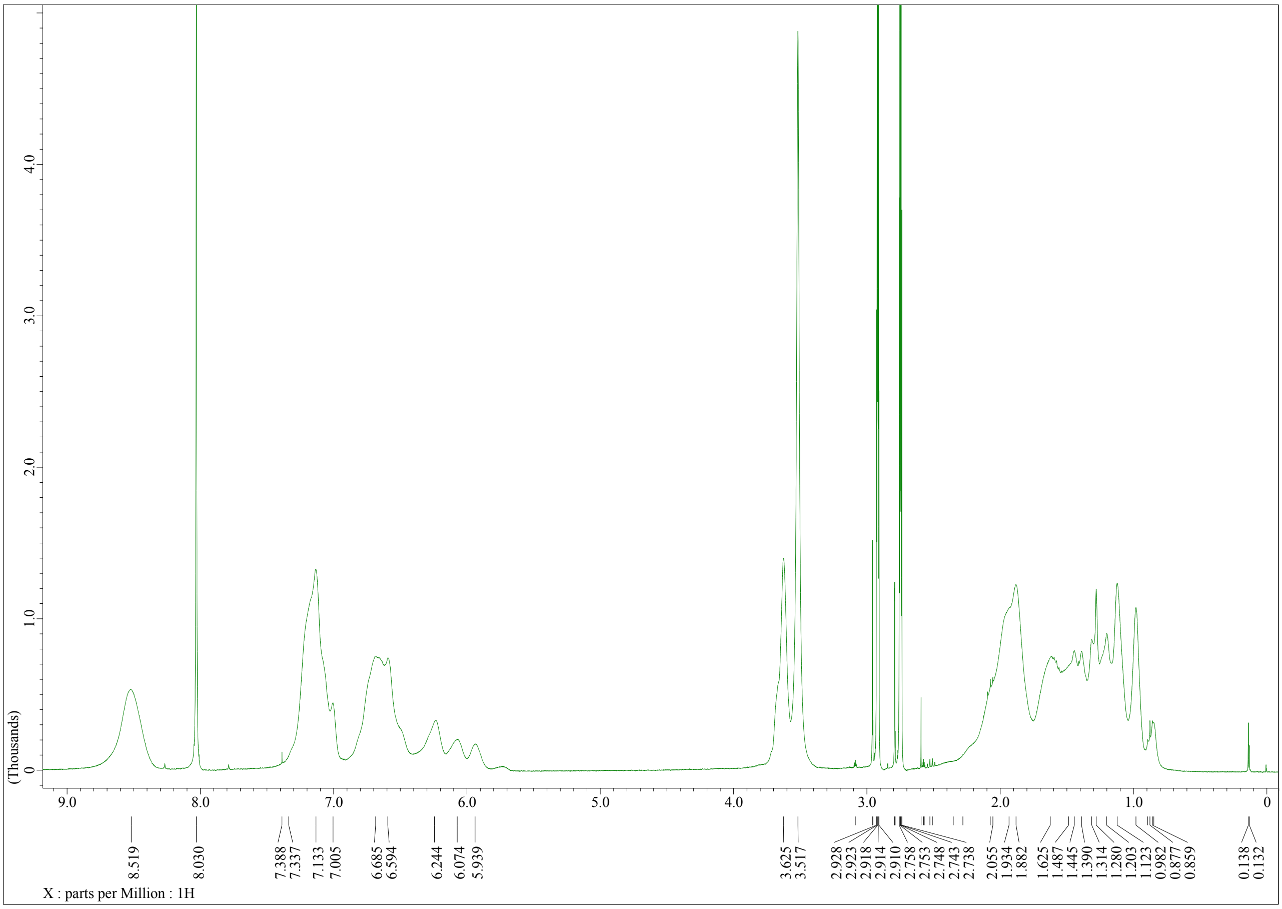

Figure S5. ^{1}H-NMR chart of PMMA-*b*-PVCa-*b*-PSt (*d*-DMF).

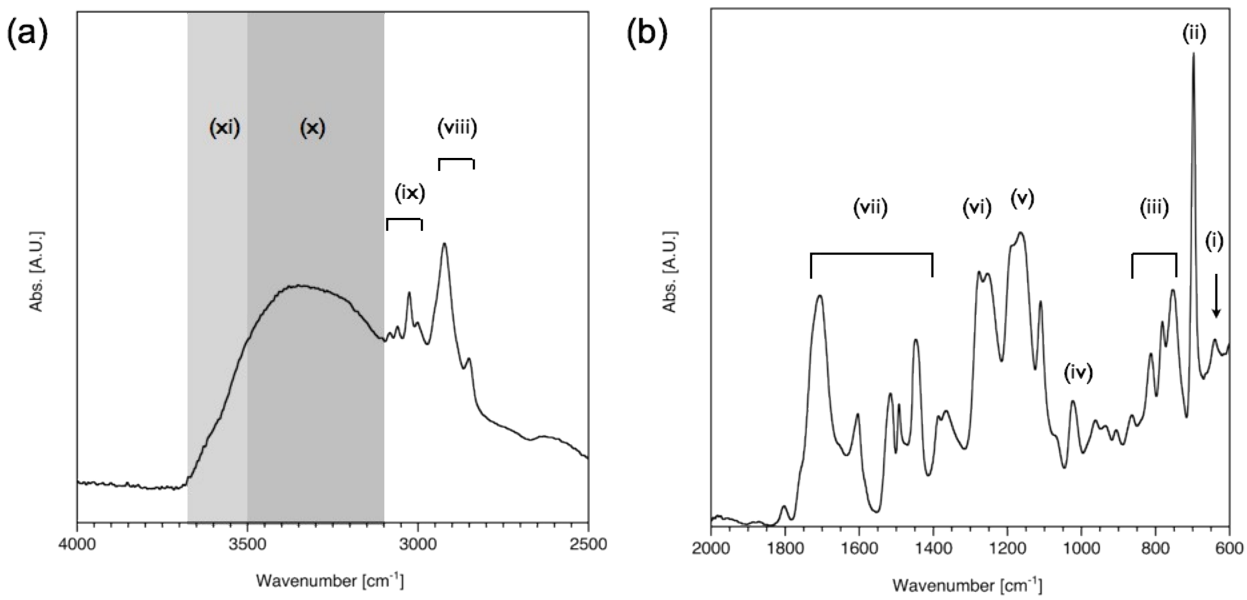

Figure S6. FT-IR spectra of PMMA-*b*-PVCa-*b*-PSt triblock copolymer ranging from 2500 cm^{-1} to 4000 cm^{-1} and from 600 cm^{-1} to 2000 cm^{-1}, respectively. Absorption attributed to the aromatic rings of styrene and catechol ($\delta_{C-H(styrene)}$ 700 cm^{-1}(ii), $\delta_{C-H(catechol)}$ 780−960 cm^{-1}(iii), $\delta_{C-H(aromatic)}$ 1,110 cm^{-1}(iv), ν_{C-C} 1,400^{-1}-1,600 cm^{-1}(vii)), the C−O vibration of catechol (ν_{C-O} 1,280 cm^{-1}(vi)), and free (ν_{O-H} 3,480−3,600 cm^{-1}(xi)) and hydrogen-bonded O−H groups (ν_{O-H} 3,125−3,480 cm^{-1}(x)) were clearly observed. From these spectra, formation of OH groups were confirmed.
